# Equine glucagon-like peptide-1 receptor physiology

**DOI:** 10.7717/peerj.4316

**Published:** 2018-01-29

**Authors:** Murad H. Kheder, Simon R. Bailey, Kevin J. Dudley, Martin N. Sillence, Melody A. de Laat

**Affiliations:** 1 Science and Engineering Faculty, Queensland University of Technology, Brisbane, Queensland, Australia; 2 Faculty of Veterinary and Agricultural Sciences, University of Melbourne, Melbourne, Victoria, Australia; 3 Institute for Future Environments, Queensland University of Technology, Brisbane, Queensland, Australia

**Keywords:** Equine metabolic syndrome, GLP-1 receptor, Horse, Insulin, Incretin, Laminitis

## Abstract

**Background:**

Equine metabolic syndrome (EMS) is associated with insulin dysregulation, which often manifests as post-prandial hyperinsulinemia. Circulating concentrations of the incretin hormone, glucagon-like peptide-1 (GLP-1) correlate with an increased insulin response to carbohydrate intake in animals with EMS. However, little is known about the equine GLP-1 receptor (eGLP-1R), or whether GLP-1 concentrations can be manipulated. The objectives were to determine (1) the tissue localisation of the eGLP-1R, (2) the GLP-1 secretory capacity of equine intestine in response to glucose and (3) whether GLP-1 stimulated insulin secretion from isolated pancreatic islets can be attenuated.

**Methods:**

Archived and abattoir-sourced tissues from healthy horses were used. Reverse transcriptase PCR was used to determine the tissue distribution of the eGLP-1R gene, with immunohistochemical confirmation of its pancreatic location. The GLP-1 secretion from intestinal explants in response to 4 and 12 mM glucose was quantified *in vitro*. Pancreatic islets were freshly isolated to assess the insulin secretory response to GLP-1 agonism and antagonism *in vitro*, using concentration-response experiments.

**Results:**

The eGLP-1R gene is widely distributed in horses (pancreas, heart, liver, kidney, duodenum, digital lamellae, tongue and gluteal skeletal muscle). Within the pancreas the eGLP-1R was immunolocalised to the pancreatic islets. Insulin secretion from pancreatic islets was concentration-dependent with human GLP-1, but not the synthetic analogue exendin-4. The GLP-1R antagonist exendin 9-39 (1 nM) reduced (*P* = 0.08) insulin secretion by 27%.

**Discussion:**

The distribution of the eGLP-1R across a range of tissues indicates that it may have functions beyond insulin release. The ability to reduce insulin secretion, and therefore hyperinsulinemia, through eGLP-1R antagonism is a promising and novel approach to managing equine insulin dysregulation.

## Introduction

Hyperinsulinemia can cause digital lamellar failure, which results in laminitis in horses (de Laat et al., 2010). The drivers for post-prandial hyperinsulinemia in horses are incompletely understood, but incretin hormone concentrations positively correlate with an increased insulin response to carbohydrate intake in predisposed breeds (Bamford et al., 2015). Circulating concentrations of the principal incretin hormone, glucagon-like peptide-1 (GLP-1), are lower in horses, than humans, as is their capacity to supplement post-prandial insulin secretion (Chameroy et al., 2016; Wu, Rayner & Horowitz, 2016). However, active GLP-1 is increased in insulin-dysregulated ponies, compared to normal ponies, and likely plays a role in metabolic dysfunction (de Laat, McGree & Sillence, 2016).

Despite recent studies demonstrating the potential importance of GLP-1 in horses, particularly with respect to managing insulin dysregulation and laminitis (Bamford et al., 2015; Chameroy et al., 2016; de Laat, McGree & Sillence, 2016), little is known about the equine GLP-1 receptor (eGLP-1R). In addition to augmenting insulin release, GLP-1 promotes the proliferation and survival of pancreatic β-cells and also has extra-pancreatic effects (Egan et al., 2003; Verges, Bonnard & Renard, 2011). If this multi-functionality also occurs in horses it is likely that the interpretation of incretin action *in vivo* is complicated by GLP-1 signalling at extra-pancreatic sites, such as the intestine (Campbell & Drucker, 2013). However, the distribution of the eGLP-1R has not been described and addressing this knowledge gap was the first objective of this study.

Reducing hyperinsulinemia is a treatment goal for horses with insulin-associated laminitis. Given the obvious difficulties associated with inhibiting insulin action, research attention needs to focus on potential mechanisms for reducing insulin secretion. Thus, the other objectives of this study *in vitro* were to measure (1) small intestinal GLP-1 secretion in response to glucose and (2) equine pancreatic islet insulin secretion in response to GLP-1, and whether this could be attenuated with a GLP-1 receptor antagonist.

## Materials and Methods

### eGLP-1R gene expression

For gene expression studies, archived samples that had been stored at −80 °C after being collected and snap-frozen using liquid nitrogen immediately following the euthanasia of nine healthy, Standardbred horses (*Equus caballus*, <15 years old) were used (The University of Queensland animal ethics approval number: SVS/013/08/RIRDC), in addition to abattoir sourced specimens from twelve horses. The collection of all abattoir specimens was approved (Queensland University of Technology tissue use approval number: 1400000039) and all horses were examined by a veterinarian prior to euthanasia. The samples included pancreas, left ventricle of the heart, kidney, digital lamellae, tongue, skeletal muscle and duodenum. Tissue samples (50–100 mg) were prepared for total RNA extraction either by being pulverised with a hammer (lamellae) or homogenised (all other tissues; Omni International, Kennesaw, GA, USA). Trizol reagent (1 mL/100 mg tissue) was used to extract the RNA according to the manufacturer’s instructions (Invitrogen, Scoresby, Vic, Australia) and genomic DNA was eliminated with RNAse-free DNAse I (Invitrogen, Scoresby, Vic, Australia). The RNA concentration and integrity were determined with a 2100 Bioanalyzer (Agilent Technologies, Santa Clara, CA, USA) prior to cDNA synthesis.

The Tetro cDNA Synthesis Kit (Bioline, Alexandria, NSW, Australia) was used to synthesise cDNA from Dnase-treated RNA. Each reaction (20 µL) contained 1 µg of cDNA, 1 µL Oligo (dT)_18_, 4 µL of 5× reverse transcription buffer, 1 µL RiboSafe Rnase inhibitor, 1 µL Tetro reverse transcriptase (200 µg/µL) and DEPC-treated water. The cDNA was stored at −80 °C until polymerase chain reaction (PCR) analysis. The eGLP-1R primer (forward: 5′-AGCGCATCTTCAGGCTCTAT-3′, reverse: 5′-GGGATGAGTGTCAGTGTGGA-3′) was designed using Primer-BLAST software (Ye et al., 2012) and primer concentration (250 nm) and PCR conditions (below) were optimised prior to use. No template, negative controls containing water instead of cDNA were included. The selected reference gene was GAPDH (GenBank accession number: AF157626).

After optimisation, PCR was performed using the One*Taq* Hot Start Quick-Load 2×  Master Mix protocol (New England BioLabs, Ipswich, MA, USA). Each 25 µL reaction contained 50 ng cDNA, 12.5 µL One*Taq* Master Mix Buffer, 10 pM/µL of each primer (forward and reverse), and 10.5 µL deionised water (dH_2_O). The PCR cycle protocol involved 94 °C initial denaturation for 30 s, 94 °C denaturation for 30 s, 60 °C annealing for 30 s, 72 °C extension for 15 s and 72 °C final extension for 5 min. After cycling, the PCR products were visualised with 2% agarose gel electrophoresis. The PCR products were purified using an ISOLATE II PCR and Gel Kit (Bioline, Alexandria, NSW, Australia) and sequenced using a standard protocol on an ABI 3500 sequencing platform (Applied Biosystems, Carlsbad, CA, USA). Sequencing confirmed amplification of the desired target and reference genes.

### eGLP-1R immunohistochemistry

Immunohistochemistry was undertaken to examine the immunolocalisation of the GLP-1 hormone, and also separately of the eGLP-1R, in the pancreas. Following harvest at the abattoir, fresh pancreatic tissues from four horses were immediately placed in 10% formalin, prior to embedding in paraffin and cutting into 5 µm thick sections. Sections were deparaffinised with xylene and then rehydrated through decreasing concentrations of ethanol (100, 95 and 70% for 1 min each) to water. Slides were then incubated in antigen retrieval solution (Target Retrieval Solution; DakoCytomation Inc., CA, USA) at 95 °C for 30 min and quenched with 0.3% hydrogen peroxide. After blocking with normal horse serum (from a fasted Standardbred horse with very low levels of serum GLP-1), the primary antibodies (GLP-1 (7-36) rabbit anti-human polyclonal; Merck, Kenilworth, NJ, USA; dilution 1:50 and GLP-1 receptor rabbit anti-human polyclonal; Lifespan Biosciences, Seattle, WA, USA; LS-A1208; dilution 1:50 [20 ug/mL]) were applied separately to duplicate sections and incubated overnight in a moist environment at 4 °C. Although not equine-specific, the immunogen sequence identity of the GLP-1 receptor antibody to *Equus caballus* was 100%. The antibody has no homology with other proteins, except weakly (50%) with the ubiquitous protein PRPSAP2, thus cross reactivity is unlikely. The slides were then washed and incubated at room temperature for 2 h with secondary antibody (mouse anti-rabbit HRP-conjugated; Santa Cruz Biotechnology, Dallas, TX, USA). Staining was developed using DAB substrate-chromogen solution (Sigma-Aldrich, Castle Hill, NSW, Australia) and the slides were counterstained with hematoxylin. Slides were then dehydrated using alcohol and xylene and mounted using DPX mounting medium (Sigma-Aldrich, Castle Hill, NSW, Australia).

### Equine GLP-1 secretion *in vitro*

Horse duodenal tissue (10 cm) was collected 30–50 cm distal to the pylorus immediately within 10 min of euthanasia of three, mixed-breed horses (at a local abattoir). The tissues were rinsed with ice-cold saline (0.9%, Baxter Healthcare, Old Toongabbie, NSW, Australia) and placed in ice-cold buffer (135 mM NaCl, 5 mM KCl, 1 mM MgCl_2_, 1.8 mM CaCl_2_, 20 mM Hepes, and 0.05% (W/V) BSA at pH 7.4) on ice for 5 min. Smaller explants (5 mm × 5 mm) of mucosal tissue were then warmed to 30 °C in buffer (as above but with oxygenation (95% O_2_/5% CO_2_)) over 10 min. Individual explants were then removed and placed immediately into the wells of a 96 well reaction plate, and incubated in triplicate in 0.8 mL of buffer containing 0, 4 or 12 mM glucose for 30 min while agitating the plate every 5 min. The glucose concentrations of 4 and 12 mM were selected to approximate fasted and markedly elevated equine blood glucose concentrations, respectively (Bamford et al., 2015; de Laat, McGree & Sillence, 2016). Following the incubation, the explants were removed and blotted dry before being weighed and discarded. The buffer was collected separately, snap-frozen and stored at −80 °C until analysed for active GLP-1 concentration with an ELISA (MPEZGLPHS35K, Merck Millipore, Billerica, MA, USA) validated (de Laat, McGree & Sillence, 2016) for use in horses (inter-assay CV 6.8%). Absorbance was measured spectrophotometrically at 450 nm (GloMax; Promega, WI, USA). The active GLP-1 concentration was corrected for the weight (mg) of the intestinal tissue explant used in each reaction/well.

### Stimulation of pancreatic islet eGLP-1Rs

Equine pancreatic islets were isolated from freshly obtained pancreas (following euthanasia of a further five healthy horses at a local abattoir, six replicates were performed per horse) as described previously (Kheder et al., 2017). Briefly, the islets were isolated using collagenase (11213857001; Roche, Mannheim, Germany) digestion of the tissue and separation of the islets through a density gradient (Histopaque 1.1 g/mL; Sigma Aldrich, Castle Hill, NSW, Australia). The freshly isolated islets from each horse (>80% live islets) were examined microscopically and then used immediately in a series of concentration–response experiments to determine insulin responses to a human peptide fragment (7-37 amide) of GLP-1 (G9416; Sigma Aldrich, Castle Hill, NSW, Australia) and a synthetic GLP-1 agonist (exendin-4, E7144; Sigma Aldrich, NSW, Australia) that has been demonstrated to be insulinogenic in other species (Kim et al., 2016). Separate aliquots were used for each experiment. The insulin secretion in each experiment was corrected for the basal insulin secretion and also normalised to protein concentration. Finally, a human GLP-1R antagonist (exendin 9-39, E7269; Sigma Aldrich, Castle Hill, NSW, Australia) was used to determine if any positive insulin responses to the agonists could be inhibited. Equine-specific products were not available.

Experiment one: Stimulation experiments using GLP-1 (7-37)were performed using aliquots of pancreatic islets (1 mL; 2.75 mM glucose) pre-incubated for 10 min prior to the addition of GLP-1 at a range of concentrations (1 × 10^−11^, 1 × 10^−10^, 1 × 10^−9^, 1 × 10^−8^ and 1 × 10^−6^ M). Each concentration was tested in triplicate over an incubation period of 60 min at room temperature (RT), before centrifugation (RT; 7 min; 16,400× g) to separate the supernatant from the cells. The incubation conditions have been optimised previously (Kheder et al., 2017). The supernatant was immediately frozen and stored at −80 °C ready for insulin analysis with a validated equine ELISA kit (Mercodia, Uppsala, Sweden; inter-assay CV 12.3%). The islets were solubilised in extraction buffer (PIE89901; Thermo Fisher Scientific, Scoresby, Vic, Australia) with a protease inhibitor cocktail (1:100 P8340; Sigma Aldrich, Castle Hill, NSW, Australia). The protein concentration of each replicate was determined (in triplicate; CV < 5%) using the bicinchoninic acid assay (PIE22323; Pierce, Rockford, IL, USA). Absorbance for both assays was measured spectrophotometrically (GloMax; Promega, WI, USA). Subsequently, the experiment was repeated in a subset of samples with a dipeptidyl peptidase-IV (DPPIV) inhibitor added (Diprotin B at 20 µM; BioVision, CA, USA), and the results compared. The enzyme DPPIV degrades GLP-1 (Baggio & Drucker, 2007).

Experiment two: The first experiment was repeated using increasing concentrations of the synthetic GLP-1 agonist exendin-4 (1 × 10^−13^, 1 × 10^−12^, 1 × 10^−11^, 1 × 10^−10^ and 1 × 10^−8^). Due to the improved performance of the assay when the DPPIV inhibitor was used in experiment one, all of the incubations in the second experiment contained Diprotin B. Islets from only three horses were measured due to unacceptably high islet death during incubation in two samples.

Experiment three: The ability of exendin 9-39 to antagonise GLP-1-stimulated insulin production was assessed under the same incubation conditions described for experiments one and two. The GLP-1 (7-37)was used at its EC_50_ of 1 nM and exendin 9-39 was used at the same concentration (Gault et al., 2003). The antagonist was added to the incubation media 10 min prior to the addition of the islets. Due to a technical problem, islets from only four of the five horses were tested with exendin 9-39.

Experiment four: Considering the ability of GLP-1 to stimulate β-cell proliferation, the islets were analysed following experiment three for evidence of cellular proliferative and apoptotic activity. Caspase 3 and proliferating cell nuclear antigen (PCNA) were used as markers of apoptosis and proliferation, respectively. Colorimetric equine-specific ELISA kits (MyBioSorce, San Diego, CA, USA) were used to determine caspase 3 and PCNA concentrations (mean assay CVs < 10%). Absorbance was measured spectrophotometrically at 450 nm (VersaMax; Molecular Devices, CA, USA).

### Statistical analyses

The data were normally distributed according to a Shapiro–Wilk test, except the PCNA data. The GLP-1 released from the intestinal explants in the absence of glucose was subtracted from the amount secreted at the two glucose concentrations tested, and these two values were compared using a paired *t*-test. The insulin concentration in islet samples containing GLP-1, with and without the antagonist, as well as the caspase 3 concentrations from the islet extracts, were also compared using a paired *t*-test. The PCNA data were examined with a Wilcoxon signed rank test and are reported as median [IQR]. All other data are reported as mean ± s.e.m. Significance was set at *P* < 0.05 with trends reported at *P* < 0.1. The EC_50_ were calculated with Prism 7.0 (GraphPad software, La Jolla, CA, USA) and the remaining data analyses were performed with SigmaPlot v.13 (Systat software, San Jose, CA, USA).

## Results

### eGLP-1R gene expression

**Figure 1:**
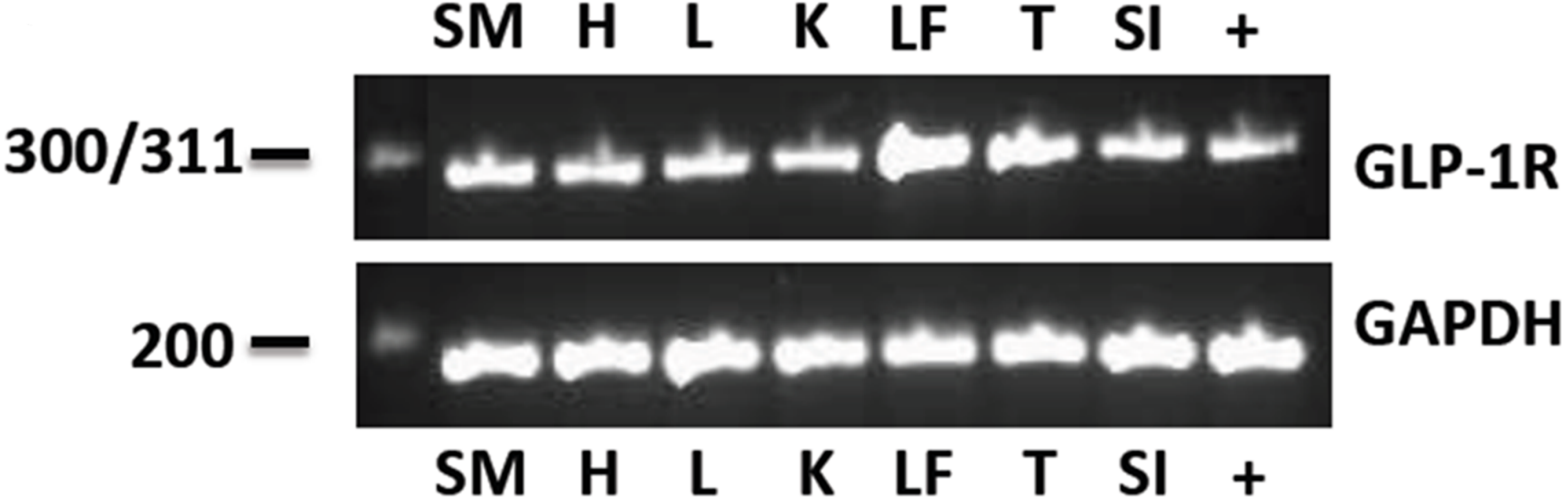
The eGLP-1R gene transcript was amplified in the pancreas (+), gluteal skeletal muscle (SM), heart (H), liver (L), kidney (K), digital lamellae of the left fore foot (LF), tongue (T) and duodenum (SI). The reference gene GAPDH was amplified equally in all tissues.

The eGLP-1R gene was consistently expressed in the pancreatic tissue from all horses examined. The sequencing data demonstrated 98–100% sequence identity to the *Equus caballus* GLP-1R mRNA sequence available from GenBank (XM_014734596). The pancreas was subsequently used as a positive control for the electrophoresis of the PCR products from the other tissues. The eGLP-1R was expressed in all of the tissues tested: the heart, liver, kidney and duodenum, digital lamellae, tongue and gluteal skeletal muscle (Fig. 1). The GAPDH gene transcript was also amplified in all tissues (Fig. 1).

### eGLP-1R immunohistochemistry

**Figure 2:**
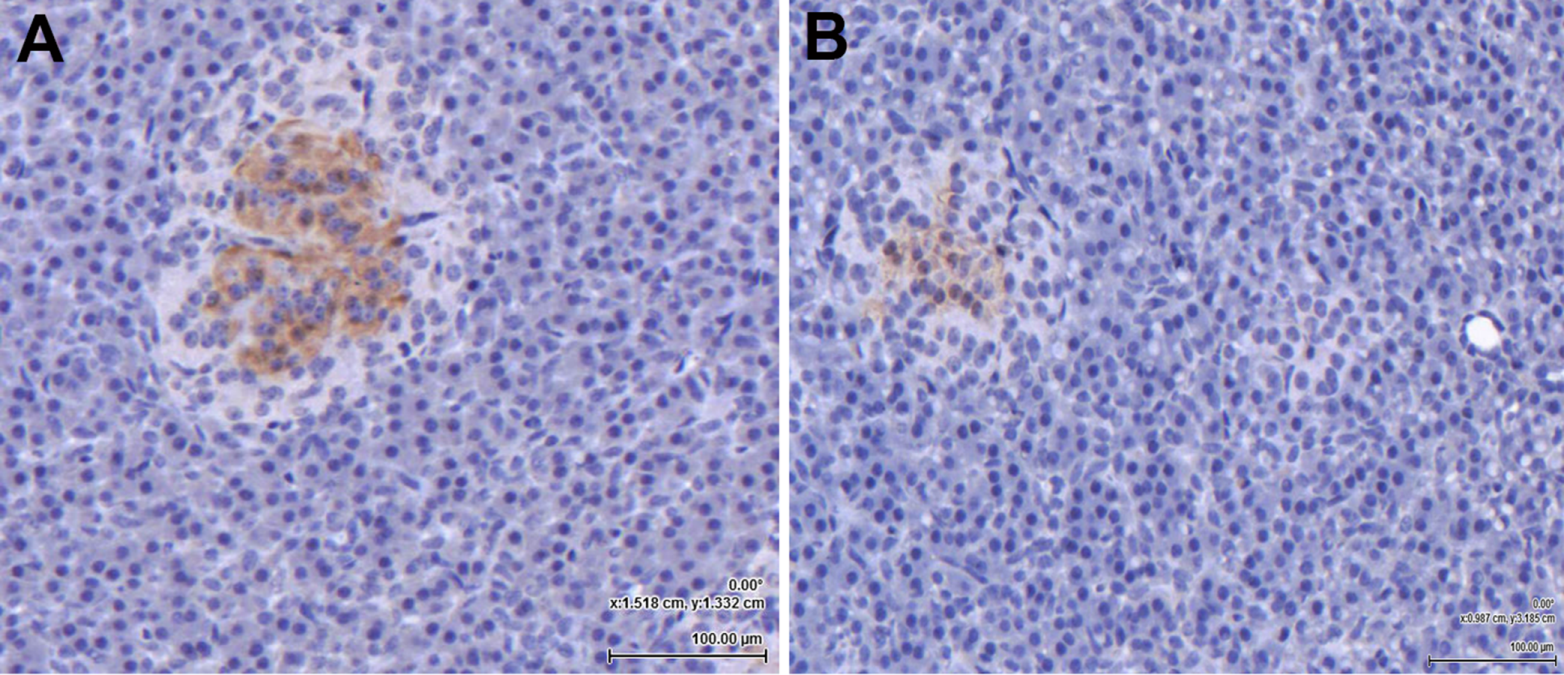
The equine GLP-1 receptor (eGLP-1R) is expressed in the pancreas. Both GLP-1 hormone (A) and the eGLP-1R (B) were immunolocalised during separate experiments to be within the islets using immunohistochemistry. Bar = 100 µm.

Following amplification of the eGLP-1R gene transcript in the pancreas, the location of the receptor within the pancreatic tissue was examined immunohistochemically. The GLP-1 hormone (Fig. 2A) and the eGLP-1R (Fig. 2B) were separately immunolocalised to be within the pancreatic islets. There was no evidence of the GLP-1 hormone or the eGLP-1R in other parts of the pancreas.

### Equine GLP-1 secretion *in vitro*

**Figure 3:**
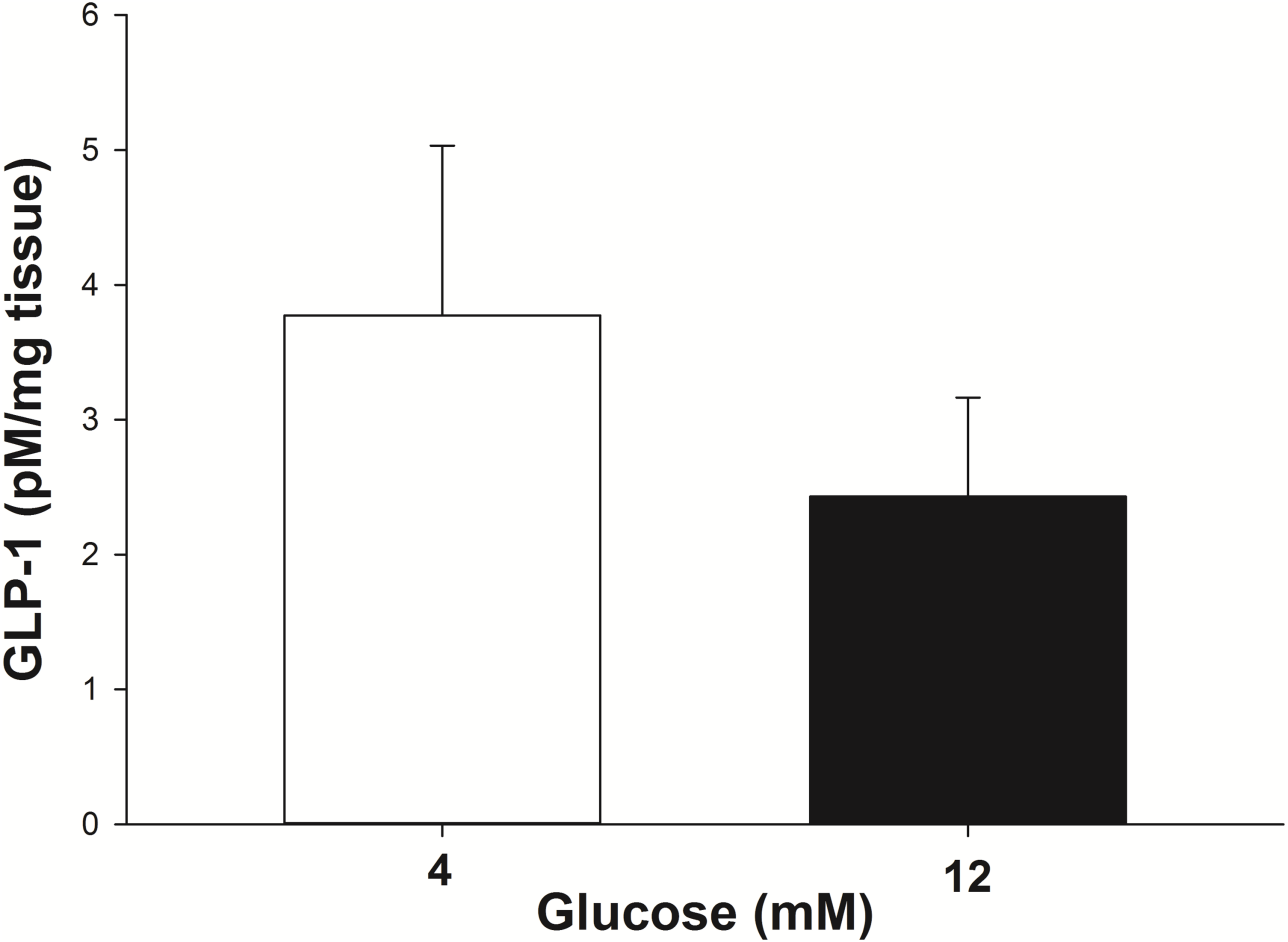
GLP-1 secretion by equine intestinal explants in response to glucose. The mean (±s.e.m.) GLP-1 concentration reached following the incubation of intestinal explants for 30 min in the presence of two glucose concentrations. GLP-1 secretion tended to be greater (*P* = 0.07) when the explants were stimulated with 4 mM glucose, compared to 12 mM glucose. The data were individually corrected for the incidental secretion when the explant was incubated without glucose.

The GLP-1 secretion from the intestinal explants under glucose stimulation (4 mM and 12 mM) was corrected for the incidental release when explants from the same animal were incubated with no glucose. At 4 mM glucose the explants secreted 3.8 ± 1.2 pM/mg of tissue of GLP-1 into the incubation media in 30 min (Fig. 3). The amount of GLP-1 secreted (per mg of tissue) trended toward being less (*P* = 0.07) when the explants were stimulated with 12 mM glucose (2.4 ± 0.75 pM/mg of tissue in 30 min).

### Stimulation of pancreatic islet eGLP-1Rs

**Figure 4:**
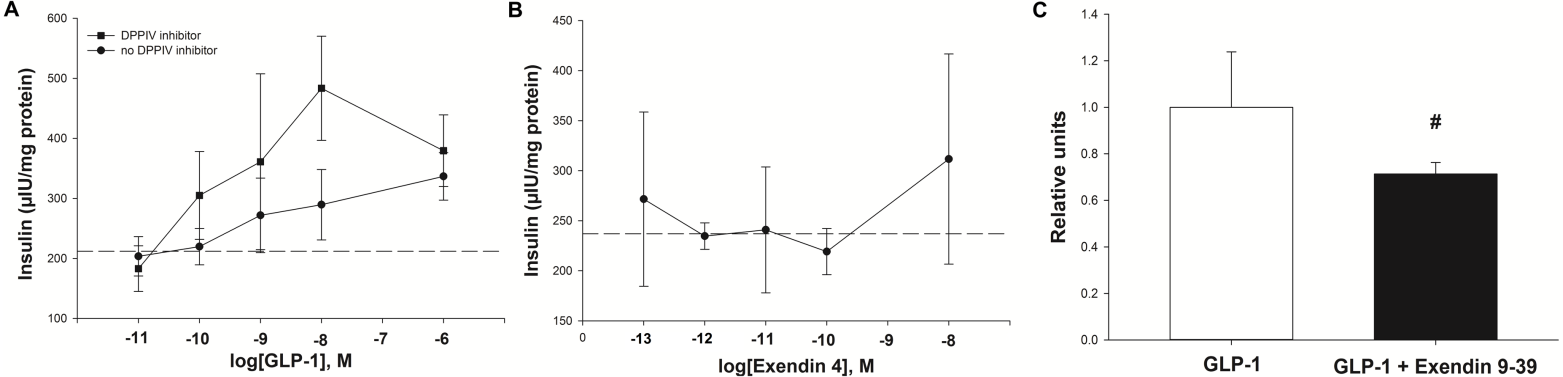
Insulin secretion by isolated pancreatic islets in response to GLP-1 agonists and an antagonist. (A) Insulin secretion was concentration-dependent with GLP-1 (7–37), and was higher in the presence of a DPPIV inhibitor. The basal insulin secretion (at 2.75 mM glucose) in the absence of GLP-1 is shown by the dashed line. (B) The synthetic GLP-1 analogue exendin-4 did not stimulate an insulin response from the isolated equine pancreatic islets. (C) The insulin release stimulated by GLP-1 (at the estimated EC_50_ of 1 nM) tended to be lower (27%, *P* = 0.08) in the presence of the antagonist exendin 9-39 (1 nM).

Experiment one: Insulin was secreted in a concentration-dependent manner in response to GLP-1 (Fig. 4A). The maximum insulin concentration (Cmax) recorded after the 60 min incubation occurred at a GLP-1 concentration of 10 nM in the presence of the DPPIV inhibitor. The GLP-1 Cmax was 484 µIU/mg of protein in the presence of the DPPIV inhibitor, and 336 µIU/mg of protein without the DPPIV inhibitor (Fig. 4A). Furthermore, the GLP-1 EC_50_ (in the presence of a DPPIV inhibitor) was estimated to be 1 nM. The insulin secretion in the absence of GLP-1 (213 ± 58 µIU/mg protein over 60 min) was consistent with that expected in response to the glucose concentration contained in the incubation medium (Fig. 4, dashed line), based on optimisation experiments.

Experiment two: In contrast to human GLP-1 7-37, the synthetic analogue exendin-4 did not elicit an insulin response from the islets (Fig. 4B), above the baseline (237 ± 61 µIU/mg protein) at any concentration tested. Accordingly, exendin-4 was not tested in conjunction with the antagonist, exendin 9-39.

Experiment three: The amount of insulin released by individual explants in the presence of GLP-1 at its estimated EC_50_ of 1 nM showed a statistical trend for being reduced (by 27%; *P* = 0.08) in the presence of 1 nM exendin 9-39 (Fig. 4C).

Experiment four: There was no significant difference in caspase 3 concentrations between GLP-1-stimulated islets when they were incubated with and without exendin 9-39 (45.8 ± 1.5 pM and 47.8 ± 1.19 pM, respectively). Further, the PCNA results were similar with no significant difference between stimulated and inhibited samples (2.5 [2.5–4.2] ng/mL and 3.7 [2.6–5.1] ng/mL, respectively).

## Discussion

Despite data *in vivo* suggesting that a functional GLP-1 receptor exists in horses, the current study is the first to demonstrate the location/s of the eGLP-1R. As is the case in other species, where the GLP-1 receptor is highly expressed on the β-cells of the pancreatic islets (Doyle & Egan, 2007; Holst, 2007), the eGLP-1R was immunolocalised within the islets of the pancreas. This finding is also consistent with previously published studies *in vivo* which have reported an entero-endocrine role for GLP-1 in augmenting insulin secretion in the horse, as occurs in many species (Baggio & Drucker, 2007; Bamford et al., 2015). The results of the present study not only demonstrate eGLP-1R gene expression in the pancreas, but further confirmed its presence using immunological techniques, which enabled localisation of the receptor to the pancreatic islet. The β-cell location of the eGLP-1R is entirely consistent with its only described function in horses to date: the augmentation of post-prandial insulin release beyond that stimulated by glucose (de Laat, McGree & Sillence, 2016).

In rodents and humans the G-protein coupled GLP-1 receptor is widely distributed across many tissues including the heart (myocytes and vasculature), kidney, lungs, intestine and the brain (Campbell & Drucker, 2013; Heppner et al., 2015; Verges, Bonnard & Renard, 2011). Despite vigorous ongoing research a precise function for the receptor has not necessarily been elucidated for each of its extra-pancreatic locations. The fact that the eGLP-1R was found in all of these tissues in the horse provides further support for the notion that the GLP-1 receptor has numerous functions beyond insulin release. Currently, the role of GLP-1 outside of the pancreas in horses is entirely unknown. The potential intestinal effects may have some bearing on equine metabolic dysfunction, where some effect on satiety and gastric emptying rate is likely based on data from studies in humans and other animals (Asmar & Holst, 2010; Martin, Mani & Mani, 2015). However, the function of the eGLP-1R in tissues remote to the entero-insular axis may be unrelated to metabolic disease.

The identification of the eGLP-1R in the digital lamellae is worth discussing. Laminitis, a major sequela to EMS in horses, manifests as separation of the lamellae from the pedal bone (McGowan, 2010). The pathophysiological mechanisms linking insulin dysregulation with lamellar failure are not currently known. It has been demonstrated that laminitis development is associated with alterations in cellular proliferative and apoptotic activity within the lamellar tissue (de Laat et al., 2013a). It is also known that GLP-1 signalling can induce cell proliferation and inhibit apoptosis in pancreatic β cells in other species (Campbell & Drucker, 2013). While evidence of these effects was not detected in the equine islet incubations in the current study, it is likely that these processes were not significantly underway within the short time frame of this study *in vitro*. It is also noteworthy that the intracellular signalling pathways triggered following activation of the GLP-1 receptor are common to other receptors that have been suggested to be involved in laminitis pathophysiology, such as the IGF-1 receptor (Campbell & Drucker, 2013; de Laat et al., 2013b). Further, an interdependent relationship between IGF-1 receptor signalling and GLP-1 action has been shown in the pancreas of rodents (Cornu et al., 2010; Cornu & Thorens, 2009). Thus, it is feasible that GLP-1 may play a direct role in laminitis pathophysiology, and this hypothesis requires investigation.

Beyond the pancreas, the actions of GLP-1 that have been elucidated most clearly are those on the cardiovascular system where, although the mechanism/s appear to be complex, the hormone is reported to support cardiovascular function, and may be a beneficial therapy for ischaemic heart disease (Ussher & Drucker, 2014). The eGLP-1R was expressed in the left ventricle of the heart, indicating that there may be a cardiovascular role for GLP-1 in horses as well. Equine cardiovascular disease is not uncommon and has recently been reported as a condition that is increasingly likely to be treated, although the prognosis for long-term resolution remains poor (Sleeper, 2017). Investigation of the potential for incretin-based therapies to be useful in equine heart disease has never been suggested, but the data from the current study suggest that further work in this area may be rewarding.

GLP-1 is produced by the L cells in the intestine (Mortensen et al., 2003). Peak post-prandial plasma GLP-1 concentrations were found to be higher in insulin-dysregulated ponies than in normal ponies (Bamford et al., 2015; de Laat, McGree & Sillence, 2016). Thus, GLP-1 secretion from the intestine of insulin-dysregulated animals may be greater than was demonstrated in the current study *in vitro* using tissue from healthy horses. This is in contrast to the situation in humans with type 2 diabetes mellitus (T2DM), where GLP-1 secretion is impaired (Bauer & Duca, 2016). Similarly, incubation of the equine intestinal explants with a high glucose concentration (12 mM) resulted in less GLP-1 release, which aligns with the situation *in vivo* during hyperglycemia in humans. However, it is also possible that the reduced GLP-1 release at 12 mM glucose may simply reflect a toxic effect of the glucose on the explant tissue. As such, further experiments utilising additional glucose concentrations would better characterize GLP-1 secretion in response to increasing glucose concentrations, and potentially establish a toxic threshold. Regardless, sustained elevations in blood glucose concentration are unlikely to occur in horses as T2DM occurs rarely in this species (Frank & Tadros, 2014). In humans, L cell density does not differ between healthy and diabetic subjects, despite the fact that GLP-1 production is reduced during T2DM (Kampmann et al., 2016). Whether there is a difference in L cell density between insulin-dysregulated and normal ponies that may contribute to the differences in circulating GLP-1 concentration remains to be determined.

This study has demonstrated that the eGLP-1R can be targeted with native human GLP-1 to manipulate insulin secretion from the pancreas. Human GLP-1 (7–37) stimulated a concentration-dependent increase in insulin secretion that could be partially inhibited by exendin 9-39, a potent and selective peptide-based exogenous GLP-1R antagonist (Nance et al., 2017). The estimated GLP-1 EC_50_ of 1nM in the current study is consistent with the EC_50_ reported in a similar study that used isolated pancreatic β-cells from mice (Serre et al., 1998). Studies on humans and rodents have demonstrated that exendin 9-39 blocks insulin release from the pancreas, induced by both GLP-1 and exendin-4, by decreasing basal cAMP production (Chan et al., 2014; Edwards et al., 1999; Nauck et al., 2016). Studies have also demonstrated that exendin 9-39 can reduce insulin secretion from isolated islets even when GLP-1 concentrations are not elevated, which suggests that the GLP-1 receptor may be constitutively active, and that exendin 9-39 may actually function as an inverse agonist (Calabria et al., 2012; Serre et al., 1998). Further studies are required to determine if this is true for the eGLP-1R.

Unlike GLP-1 (7-37), exendin-4 did not stimulate an insulin response from the equine pancreatic islets. This lack of efficacy in the horse may be related to structural differences between the two compounds, with exendin-4 being a longer and more complex molecule (Neidigh et al., 2001). Perhaps the synthesis of an equine-specific product would produce optimal outcomes and this approach should be pursued for future studies.

Overall, this study has provided novel data regarding the eGLP-1R that can be utilized by future studies. However, given the *in vitro* nature of the current study, it is imperative that these data be seen as a baseline to guide future work that aims to determine the likely effect of eGLP-1R agonists and antagonists/inverse agonists *in vivo*. The ability to reduce insulin secretion, and therefore hyperinsulinemia, through the use of eGLP-1R antagonism is an exciting outcome of this study which may represent a promising, and novel, approach to managing equine insulin dysregulation.

##  Supplemental Information

10.7717/peerj.4316/supp-1 Supplemental Information 1 Raw file dataset

10.7717/peerj.4316/supp-2 Supplemental Information 2 Uncut gel scan for PCR raw data

## Additional Information and Declarations

### Data Availability

The following information was supplied regarding data availability: The raw data is included in the Supplemental Files.
