# Supplementary figures and images for: Equine glucagon-like peptide-1 receptor physiology
Source: PeerJ. 2018 Jan 29;6:e4316. doi: 10.7717/peerj.4316 (PMC5793710; doi:10.7717/peerj.4316)

qlp-1new DIST. final (Raw 1-D Image)

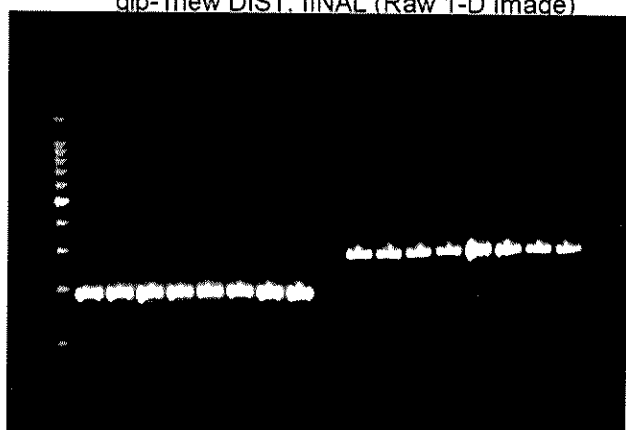

M H L K L F T S I +ve | M H L K L F T S I -ve  
-ve +ve

Supplement: Supplemental Information 2 [file peerj-06-4316-s002.pdf]
